# Navigating P2Y12 inhibition in the labyrinth of cardio-oncology care: cangrelor bridging in patients with cancer

**DOI:** 10.3389/fcvm.2024.1337957

**Published:** 2024-02-29

**Authors:** Abdelrahman Ali, Poonam Jewani, Max Bourdillon, Efstratios Koutroumpakis, Shaden Khalaf, Konstantinos Charitakis, Kara Thompson, Konstantinos Marmagkiolis, Anita Deswal, Cezar Iliescu

**Affiliations:** ^1^ Department of Cardiology, MD Anderson Cancer Center, Houston, TX, United States; ^2^ Division of Cardiology, Department of Medicine, McGovern Medical School, University of Texas, Houston, TX, United States

**Keywords:** coronary artery disease, cangrelor, thrombocytopenia, bridging, P2Y12 Inhibitors

## Abstract

Cangrelor, a potent intravenous P2Y12 platelet inhibitor, has demonstrated effectiveness in reducing ischemic events without a corresponding increase in severe bleeding during percutaneous coronary intervention, as evidenced by the CHAMPION-PHOENIX trial. Its off-label role as a bridging antiplatelet agent for patients facing high thrombotic risks who must temporarily stop oral P2Y12 inhibitor therapy further underscores its clinical utility. This is the first case series to shed light on the application of cangrelor in cancer patients needing to pause dual antiplatelet therapy for a range of medical interventions, marking it as a pioneering effort in this domain. The inclusion of patients with a variety of cancer types and cardiovascular conditions in this series underlines the adaptability and critical role of cangrelor in managing the dual challenges of bleeding risk and the need for uninterrupted antiplatelet protection. By offering a bridge for high-risk cancer patients who have recently undergone percutaneous coronary intervention and need to halt oral P2Y12 inhibitors temporarily, cangrelor presents a practical solution. Early findings indicate it can be discontinued safely 2-4 h before medical procedures, allowing for the effective reintroduction of oral P2Y12 inhibitors without adverse effects. This evidence calls for expanded research to validate and extend these preliminary observations, emphasizing the importance of further investigation into cangrelor's applications in complex patient care scenarios.

## Introduction

Cangrelor, an intravenous P2Y12 platelet inhibitor, has reduced the rate of ischemic events compared with oral P2Y12 inhibition, without causing significant increases in severe bleeding during percutaneous coronary intervention (PCI), as demonstrated by the CHAMPION-PHOENIX randomized controlled trial (RCT) (1). In addition, cangrelor has been used off-label as bridging antiplatelet therapy in patients with PCI at high thrombotic risk who require early (within 3–6 months) interruption of their oral P2Y12 inhibitor therapy (2). Advancements in cancer therapy have improved patient survival rates, leading to an increased number of cardiac procedures, such as PCI, among patients treated for cancer. However, there is limited information on the utilization of cangrelor in patients with cancer, particularly in those with thrombocytopenia (3). In this study, we present a single-center case series of cangrelor bridging in patients with cancer and recent PCI that required temporary interruption of dual antiplatelet therapy (DAPT).

## Case 1

A 69-year-old woman with a past medical history (PMH) of IgA kappa multiple myeloma (MM) with a history of autologous stem cell transplant on maintenance lenalidomide, end-stage renal disease on peritoneal dialysis, saphenous venous thrombosis, and coronary artery disease (CAD) with recent non-ST-segment elevation myocardial infarction (NSTEMI) had a PCI to the mid-left anterior descending artery (LAD) 3 months prior to presentation with melena and acute on chronic anemia (hemoglobin 5.6 g/dl). The patient was taking both clopidogrel and apixaban prior to admission and was noted to have an allergy to aspirin. On admission, both clopidogrel and apixaban were held due to concerns for gastrointestinal (GI) bleeding. The patient underwent a coronary angiogram with optical coherence tomography (OCT), which revealed suboptimal apposition of the previously implanted LAD stent (by approximately 20%). The interventional cardiology team recommended the initiation of cangrelor on day 3 of admission in anticipation of further GI evaluation. The patient underwent an upper GI endoscopy on hospital day 6 and no active bleeding was observed. Cangrelor infusion was discontinued after endoscopy, and clopidogrel was resumed with a 300 mg loading dose. The patient's apixaban was not resumed at discharge as the patient no longer had an indication for apixaban after consultation with the hematology service and also due to thrombocytopenia (39 K/μL).

## Case 2

A 55-year-old woman with metastatic gallbladder adenocarcinoma, hypertension, type II diabetes, and CAD, who underwent a PCI of a bifurcating lesion of the distal right coronary artery (RCA) extending into posterolateral and posterior descending artery (PDA) branches with two drug-eluting stents (DES) 2 months prior to the current admission, presented with biliary obstruction. The patient was on DAPT with aspirin and clopidogrel prior to admission. The last dose of clopidogrel was administered on day 2 of hospitalization, and cangrelor was initiated on day 3 with the recommendation to hold 4 h prior to the planned procedure. The patient underwent an endoscopic retrograde cholangiopancreatography (ERCP) with stent placement on day 8. Cangrelor was discontinued postprocedure, and clopidogrel was restarted 2 days post procedure with a 300 mg loading dose, followed by standard 75 mg daily dosing.

## Case 3

A 64-year-old man with relapsed diffuse large B-cell lymphoma s/p chimeric antigen receptor (CAR) *T*-cell therapy (10 days prior to presentation), atrial fibrillation, and ischemic cardiomyopathy (ICM) underwent a high-risk PCI (10 weeks prior to hospital admission) with three overlapping stents in the ostial to mid-LAD, and residual RCA chronic total occlusion, was hospitalized for fever and profound fatigue. The patient's home DAPT of aspirin and clopidogrel was initially continued during hospital stay. On hospital day 5, the patient received his last dose of clopidogrel, and cangrelor was initiated the following day in preparation for a planned endoscopic evaluation for cytomegalovirus colitis. Aspirin was discontinued 2 days after cangrelor initiation because of worsening thrombocytopenia (platelet count, 32 K/µl). Given the patient's clinical improvement, elevated cardiac risk, and altered mental status, colonoscopy was deferred, and clopidogrel was restarted without a loading dose (platelet count, 21 K/µl). He was discharged on clopidogrel monotherapy because of significant thrombocytopenia (platelet count <50 K/µl).

## Case 4

A 74-year-old woman with metastatic ovarian cancer (including pulmonary metastases and peritoneal involvement), hypertension, and CAD s/p PCI to mid-LAD (3 weeks prior to presentation) presented with small bowel obstruction. She was on DAPT with aspirin and ticagrelor prior to admission. Resumption of the patient's DAPT was complicated by a strict nothing by mouth (NPO) status. Rectal aspirin was recommended, but the patient deferred in taking it. In lieu, heparin infusion was initiated on the first day of hospitalization, and she was eventually transitioned to cangrelor on day 4 of admission, as it was unclear when the patient would be able to resume oral antiplatelet therapy. A gastrostomy tube (G-tube) was placed by interventional radiology on day 8 of the patient’s hospitalization, for which cangrelor was held 3 h prior to the procedure. DAPT was resumed the following day with a 180 mg loading dose of ticagrelor. The patient was eventually transitioned to home hospice care.

## Case 5

An 82-year-old man with a history of IgG kappa MM, AL-amyloidosis, CAD s/p coronary artery bypass graft (CABG) with left internal mammary artery (LIMA) to the LAD and saphenous vein graft (SVG) to PDA, and a recent NSTEMI treated with DES implantation to the left main coronary artery (LMCA) into the left circumflex artery (LCX) 6 weeks prior, presented with a right intertrochanteric lytic lesion. This lesion was associated with a non-displaced pathological fracture, necessitating surgical stabilization. The patient was on DAPT with aspirin and clopidogrel at home. While aspirin was continued on admission, clopidogrel was replaced with cangrelor. The procedure was performed on day 5 of hospitalization, and cangrelor was held for 2 h prior to procedure. Cangrelor infusion was resumed in the evening of the procedure, and the patient was transitioned back to clopidogrel with a loading dose of 300 mg two days later when the risk of bleeding was considered to be low by the primary team.

## Case 6

A 68-year-old man with a history of a newly diagnosed metastatic mesenteric mass to the liver, hypertension, type II diabetes, ST-segment elevation myocardial infarction (STEMI) treated with PCI to the proximal LAD 5 months earlier, and moderate aortic valve stenosis, presented to the hospital for an elective interventional radiology (IR)-guided biopsy. The patient had been on DAPT with aspirin and clopidogrel. Upon admission, aspirin was continued, clopidogrel was discontinued, and cangrelor bridging was initiated. A liver biopsy was performed on day 6 of hospitalization, and cangrelor was held 3 h prior to the procedure. The following day, clopidogrel was resumed with a 300 mg loading dose. The biopsy revealed a metastatic well-differentiated neuroendocrine tumor.

## Case 7

An 85-year-old man with a history of salivary duct carcinoma of the right lower eyelid, hypertension, and CAD with PCI to the distal RCA 6 months prior for NSTEMI was admitted for cangrelor bridging prior to excision of a malignant lesion of the eyelid. Both aspirin and clopidogrel were to be stopped in the perioperative period, given the high risk of bleeding due to extensive surgery and reconstruction within the orbit, and the risk of vision loss if bleeding occurred. On admission, periprocedural cangrelor bridging was initiated. After a 1-week washout, the patient underwent the procedure uneventfully. Aspirin and clopidogrel were resumed 2 days after the procedure (both without loading doses per request from the surgical team).

## Discussion

Patients with cancer present an elevated risk for both bleeding and thrombotic events. Managing patients who have CAD and stents in the context of cancer therapy is a complex task due to the frequent need for a shortened duration of DAPT. This is the first single-center experience with bridging antiplatelet therapy using cangrelor in patients with cancer undergoing non-cardiac interventions. Landmark RCTs such as CHAMPION-PCI, CHAMPION-PLATFORM, and CHAMPION-PHOENIX excluded patients with active cancer (1, 4, 5). Moreover, patients with cancer were not included in the BRIDGE trial, which assessed bridging antiplatelet therapy with cangrelor in patients undergoing CABG (2). The BRIDGE trial specifically consisted of two stages, an initial dose-finding phase for cangrelor, followed by a randomized, double-blind, placebo-controlled trial of 210 patients with acute coronary syndrome (ACS) or with a coronary stent receiving P2Y12 inhibitor therapy and awaiting CABG surgery. The trial demonstrated that preoperative use of cangrelor was associated with a reduction in platelet reactivity without an increase in CABG surgery–related bleeding. The median duration of cangrelor infusion was 2.8 days and median time from discontinuation to surgical incision was 3.2 h.

Expert consensus from the Society for Cardiovascular Angiography and Interventions in late 2016 did not offer recommendations for bridging with cangrelor in patients with active cancer, who may have concomitant thrombocytopenia due to the limited collective experience accumulated in the short time from FDA approval of cangrelor was June 2015 (6). However, the document stated that thrombocytopenia does not have a protective role against ischemic events, and in some specific malignancies with blood dyscrasias, it poses an increased thrombotic risk (7).

All patients in this case series had active cancer, including multiple myeloma, neuroendocrine tumor, lymphoma, gallbladder cancer, and ovarian cancer. The mean duration of cangrelor bridging was approximately 4.5 days (Table 1), and all patients except one underwent a PCI for ACS within 6–24 weeks of admission. The dose of cangrelor used for all seven patients was 0.75 μg/kg/min, which is consistent with the BRIDGE trial (2). The age range of the patients was 55–85 years old. None of the patients experienced any bleeding or thrombotic events during bridging therapy or postprocedure. Cangrelor has a very short half-life of 3–6 min. In the BRIDGE trial, it was discontinued 1–6 h before surgery. In most of our patients, we halted cangrelor 2–4 h prior to surgery or procedure based on the primary team's advice for the procedure. To date, there is only one case report in the cardio-oncology literature describing cangrelor bridging to facilitate lung biopsy in a 67-year-old patient who underwent a PCI of the mid-LAD 16 months prior to biopsy on extended DAPT therapy (3). Details of the patient's initial clinical presentation for PCI were not reported nor the rationale stated for the extended DAPT therapy. In contrast, our case series included only those patients who underwent PCI within the preceding 6 months, Several with an acute coronary syndrome indication, and required DAPT interruption.

**Table 1:** Baseline characteristics of patients receiving cangrelor.

Baseline characteristics	Case 1	Case 2	Case 3	Case 4	Case 5	Case 6	Case 7
Age (years)	69	55	64	74	82	68	85
Sex	Female	Female	Male	Female	Male	Male	Male
Baseline platelet count (K/μL)	39	383	51	256	178	222	154
CAD presentation	N-STEMI	Unstable angina	ICM	N-STEMI	N-STEMI	TSEMI	N-STEMI
Duration between index PCI and presentation (weeks)	12	8	10	3	6	20	24
Procedure	Endoscopy	ERCP	Colonoscopy	G-tube placement	Orthopedic	IR-guided biopsy	Eyelid malignant lesion excision
Duration of therapy (days)	3	5	3	4	5	5	7
Concomitant aspirin	None	Yes	Discontinued	Discontinued	Yes	Yes	None
Bleeding within 30 days	None	None	None	None	None	None	None
Ischemic within 30 days	None	None	None	None	None	None	None
Stent type	NA	Orsiro DES	Synergy DES	Synergy DES	Synergy DES	Onyx DES	NA
Number of stents	NA	2	3	1	2	NA	NA

CAD, coronary artery disease; ICM, ischemic cardiomyopathy; ERCP, endoscopic retrograde cholangiopancreatography; G-tube, gastrostomy tube; IR, interventional radiology; N-STEMI, non-ST segment elevation myocardial infarction; STEMI, ST segment elevation myocardial infarction; NA, not available; DES, drug eluting stent.

**Figure 1:**
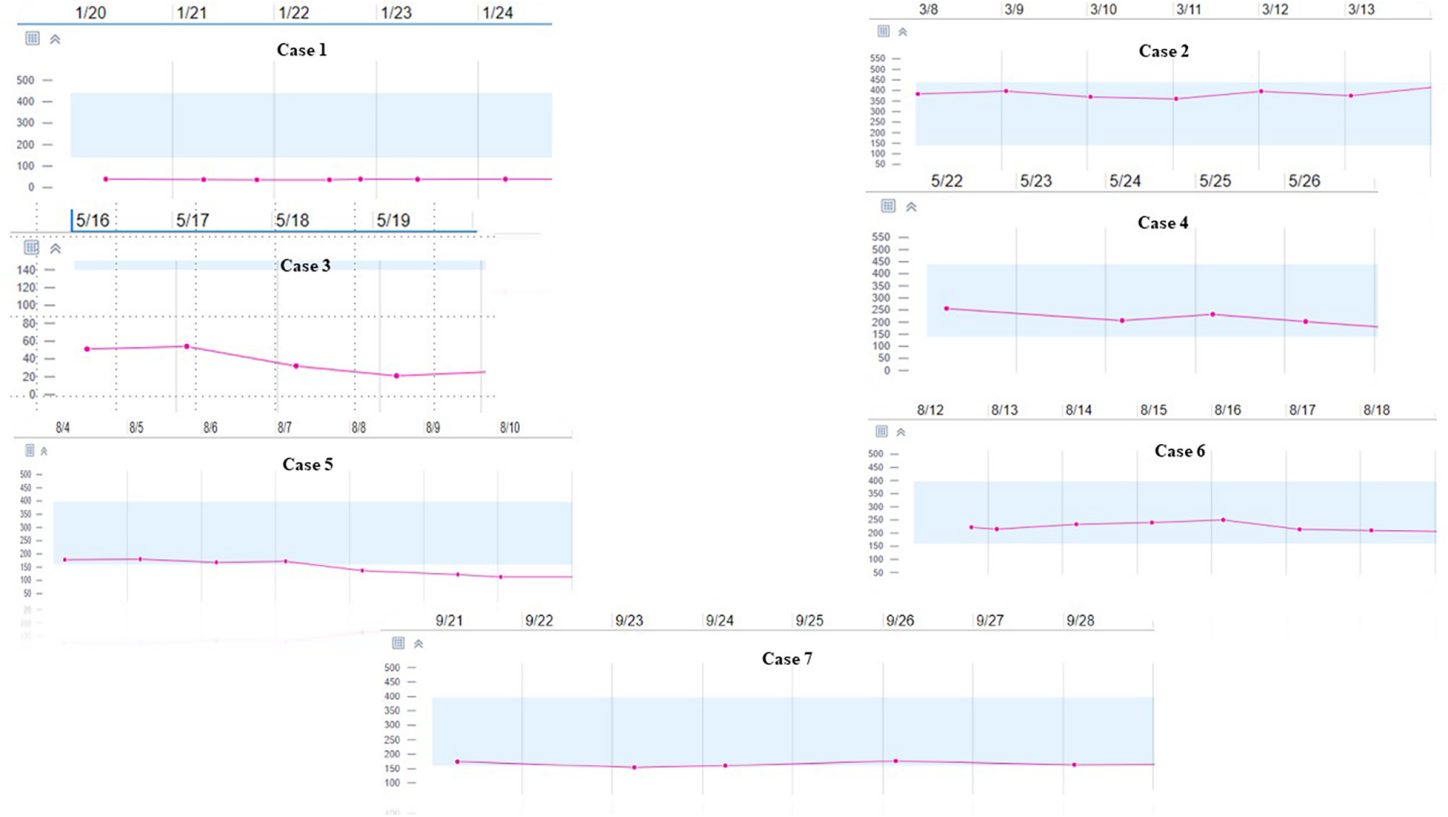
Trend of Platelet Count During Cangrelor Therapy.

A significant proportion of patients receiving cancer therapy and the majority of patients with active hematological malignancies have thrombocytopenia. Cangrelor has been used in patients with thrombocytopenia but not specifically in patients with cancer (8, 9). Cancer and therapy can actively modify both bleeding and thrombotic risks. Two of our patients had profound thrombocytopenia (cases 1 and 3; Figure 1), but neither experienced bleeding events. In case 1, clopidogrel monotherapy was restarted after cangrelor bridging, and apixaban was not resumed as detailed, while case 3 was de-escalated to clopidogrel monotherapy at discharge due to persistent thrombocytopenia with a platelet count <50 K/µl. In a pooled analysis of the CHAMPION trials, acquired thrombocytopenia (platelet count <100 K/µl) occurred in 0.8% of the study cohort (10). In addition, periprocedural use of glycoprotein IIb/IIIa inhibitors was the strongest independent predictor of acquired thrombocytopenia. There was no statistical difference in the rate of acquired thrombocytopenia between patients who were randomized to receive either clopidogrel or cangrelor. A case report by Kabadi et al. highlighted the use of cangrelor bridging therapy in the setting of left ventricular assist device implantation in a patient with refractory cardiogenic shock post-STEMI. The patient developed acute thrombocytopenia with tirofiban use (9). However, the severity of thrombocytopenia (nadir 88 × 10^3 ^/mm^3^) was milder than that observed in our cardio-oncology population.

## Conclusions

This report presents the use of an intravenous P2Y12 inhibitor for bridging high-risk cancer patients with recent PCI during temporary cessation of oral P2Y12 inhibitors. Preliminary data suggests that cangrelor can be used in patients with cancer and thrombocytopenia, and can be safely discontinued 2–4 h before the start of procedures. Most patients were reloaded with oral P2Y12 inhibitors without their experiencing any adverse outcomes. Tailored therapeutic strategies based on individual risks and procedures are critical. Larger prospective studies are required to address this knowledge gap, necessitating cardiology–oncology collaboration to enhance the care of this complex patient population.

## Data Availability

The original contributions presented in the study are included in the article/Supplementary Material, and further inquiries can be directed to the corresponding author.
